# Monitoring Nusinersen Treatment Effects in Children with Spinal Muscular Atrophy with Quantitative Muscle MRI

**DOI:** 10.3233/JND-221671

**Published:** 2024-01-02

**Authors:** Louise A.M. Otto, M. Froeling, Ruben P.A. van Eijk, Renske I. Wadman, Inge Cuppen, Danny R. van der Woude, Bart Bartels, Fay-Lynn Asselman, Jeroen Hendrikse, W. Ludo van der Pol

**Affiliations:** a Department of Neurology, UMC Utrecht Brain Center, University Medical Center Utrecht, Utrecht University, Utrecht, The Netherlands; b Department of Radiology, University Medical Center Utrecht, Utrecht University, Utrecht, The Netherlands; c Biostatistics & Research Support, Julius Center for Health Sciences and Primary Care, University Medical Center Utrecht, Utrecht University, Utrecht, The Netherlands; d Department of Neurology and Child Neurology, UMC Utrecht Brain Center, University Medical Center Utrecht, Utrecht University, Utrecht, The Netherlands; e Department of Child Development and Exercise Center, University Medical Center Utrecht, Utrecht University, The Netherlands

**Keywords:** Muscular atrophy, spinal, magnetic resonance imaging, diffusion tensor imaging, therapeutics

## Abstract

**Background:**

Spinal muscular atrophy (SMA) is caused by deficiency of survival motor neuron (SMN) protein. Intrathecal nusinersen treatment increases SMN protein in motor neurons and has been shown to improve motor function in symptomatic children with SMA.

**Objective:**

We used quantitative MRI to gain insight in microstructure and fat content of muscle during treatment and to explore its use as biomarker for treatment effect.

**Methods:**

We used a quantitative MRI protocol before start of treatment and following the 4th and 6th injection of nusinersen in 8 children with SMA type 2 and 3 during the first year of treatment. The MR protocol allowed DIXON, T2 mapping and diffusion tensor imaging acquisitions. We also assessed muscle strength and motor function scores.

**Results:**

Fat fraction of all thigh muscles with the exception of the m. adductor longus increased in all patients during treatment (+3.2%, *p* = 0.02). WaterT2 showed no significant changes over time (–0.7 ms, *p* = 0.3). DTI parameters MD and AD demonstrate a significant decrease in the hamstrings towards values observed in healthy muscle.

**Conclusions:**

Thigh muscles of children with SMA treated with nusinersen showed ongoing fatty infiltration and possible normalization of thigh muscle microstructure during the first year of nusinersen treatment. Quantitative muscle MRI shows potential as biomarker for the effects of SMA treatment strategies.

## INTRODUCTION

Hereditary proximal spinal muscular atrophy (SMA) is caused by loss of function of the survival motor neuron (*SMN)1* gene and the resulting deficiency of cellular SMN protein. SMA is characterized by severe and progressive muscle weakness and is the most frequent genetic cause of infantile death or severe impairment later in life [1]. SMN protein is ubiquitously expressed and has several important cellular functions [2]. The highly homologous *SMN2* gene ensures the production of residual amounts of full-length protein, but this is insufficient to avoid degeneration of the motor unit [3]. Alpha motor neurons are most sensitive to SMN deficiency, but SMN deficiency probably also affects the structural integrity and function of other tissues, such as muscle [4, 5].

The antisense oligonucleotide (ASO) Nusinersen modulates *SMN2* mRNA splicing and is the first approved drug for SMA. It is used to treat an increasing number of patients worldwide. After intrathecal administration, it upregulates SMN production in the spinal cord and brain [6, 7]. Randomized trials have shown that nusinersen treatment improves survival in infants and motor function in approximately half of young (symptomatic) children with SMA [8–10]. Real life data in older symptomatic patients also show treatment effects in subgroups [11, 12]. The clinical motor function scales that have been used in natural history studies to assess disease progression lack sensitivity to detect relevant changes in the shorter run [13, 14]. New, sensitive biomarkers that can differentiate responders from non-responders would therefore help to improve cost-effectiveness and reduce the patient burden that is associated with unsuccessful treatment.

Magnetic resonance imaging (MRI) has been used as an *in vivo* outcome measure in trials to measure treatment effect in patients with neuromuscular disorders such as Duchenne Muscular Dystrophy (DMD) [15–17]. We recently showed that quantitative MRI (qMRI) of thigh muscles correlates with clinical outcome measures and can detect subclinical disease progression in the course of one year in treatment-naïve adult patients with SMA [14, 18]. This MR protocol assesses the ratio of healthy muscle versus fat fraction (DIXON); microstructural changes in remaining muscle tissue using diffusion tensor imaging (DTI) and possible inflammatory changes (T2 mapping). In the current study, we used this MR protocol in a cohort of 8 young children with SMA type 2 and type 3 (age range 7–13 years) prior to and in the course of the first year of treatment (at 4th and 6th injection) to investigate if qMRI has potential as a biomarker to detect treatment effects. The data presented here contribute to our understanding of treatment effects on tissue level as qMRI detects early changes during treatment, making it a promising tool for treatment evaluation.

## MATERIAL AND METHODS

### Study population

We consecutively enrolled 8 children with genetically confirmed SMA before they started treatment with nusinersen. Four children had SMA type 2 (i.e. onset between 6–18 months and able to sit independently at any moment in life) and 4 had SMA type 3 (i.e. onset after 18 months and able to walk independently at any moment in life) and no other co-morbidities [19]. Mean age was 9.0 years (range 7.6–13.8 years). Clinical characteristics are presented in Table 1. The inclusion criterium was an age older than 6 years at the start of nusinersen treatment. From this age children can express voluntary participation to scanning procedures without sedation. In three instances, the child refused participation which resulted in a screeningfailure. Exclusion criteria were any type of invasive ventilation, orthopnea, pronounced swallowing disorders, discontinuation of nusinersen treatment during the study, or any other contra-indication for 3 Tesla MR.

**Table 1:** Patient characteristics at baseline

	Patients (*n* = 8)
Mean age in years [range]	9.0 [7.7–13.8]
Sex (M:F)	5:3
SMA type (Type 2: Type 3)	4:4
*SMN2* copy number*
3	5
4	3
Mean disease duration in months [range]	93 [69–148]
Ambulatory status (ambulant: non-ambulant)	3:5

Legend table 1: F = female; M = male. *SMN2 copy number was determined with MLPA analysis (SALSA MLPA kit P060 version B2; www.mlpa.com).

The study was approved by the local ethics committee (no. 17-226/NL61066.041.17) and conducted in accordance with codes of conduct for research in children and the Helsinki declaration (latest amendment Fortaleza, October 2013). In children under 12 years, parents gave oral and written consent. We monitored children for signs of resistance against any of the procedures. Children of 12 years and older and their parents both gave oral and written consent.

### Clinical evaluation

Two trained physiotherapists (DvdW, BB) assessed motor function with the Hammersmith Functional Motor Scale Expanded (HFMSE) before the 1st and after the 4th (i.e. 2 months after start of treatment) and 5th injection (i.e. 6 months after start of treatment). In addition to routine clinical evaluation with HFMSE, we assessed muscle strength either prior to the injection or directly following the MR examination. The same evaluator (LAMO) performed all strength measurements using the Medical Research Council (MRC) scale and hand-held dynamometry (MicroFET2; Hoggan Health Industries Inc., USA) of the quadriceps, hamstrings and adductors of both sides according standard procedures [20]. We report muscle strength as a composite MRC score of all 42 muscle groups (flexors and extensors of the neck, upper arms, wrists, fingers, hips, knees, feet and deltoids, pectoralis, supraspinatus, infraspinatus, finger abductors, hip abductor and –adductor and hallucis longus muscle) and as MRC sum score of the upper leg muscles (quadriceps, hamstrings and adductors).

### MR acquisition

Baseline scans were performed prior to the first injection with nusinersen. The second and third scan were scheduled at the 4th injection (i.e. 2 months after start of treatment) and at the 6th injection (10 months after start of treatment). We used the same 3T MR scanner (Philips Ingenia, Philips Medical Systems, the Netherlands) for all but one patient. We had to reschedule this patient to another scanner from the same vendor because it was out-of-service due to maintenance. Patients were scanned in supine position, feet-first with a 12-channel posterior coil and 16-channel anterior body coil. The image stack was centered mid-femoral, the distance from the top of the FOV to the upper limit of the femoral head was noted for each patient to ensure same positioning for follow-up scans. We designed the protocol to be short (total scan time ∼10 minutes) in order to be tolerated by young children.

The MR protocol consisted of the following three sequences; 4-point DIXON (TR/TE/ 210/2.6/3.36/4.12/4.88 ms; flip angle 10°; voxel size 6×1.5×1.5 mm^3^; no gap; 25 slices); T2 mapping (17 echoes TR/TE/*Δ*TE 4598/17/7.6 ms; flip angle 90/180°; voxel size 6×3×3 mm^3^; slice gap 6 mm; 13 slices, no fat suppression) and DTI SE-EPI (TR/TE 5000/57 ms; b-values: 0 (1), 1 (6), 10 (3), 25 (3), 100 (3), 200 (6), 400 (8) and 600 (12) s/mm^2^; voxel size 6×3×3 mm^3^; no gap; 25 slices, SPAIR and SPIR fat suppression. The MR protocol has been validated in a multicenter study on healthy controls and has been used in previous imaging studies in SMA [14, 18, 21].

### MR processing

We obtained the following quantitative parameters; fat fraction (%) from DIXON; waterT2 relaxation times (ms); mean diffusivity (MD) and the directional parameters fractional anisotropy (FA), radial diffusivity (RD) and axial diffusivity (AD) from DTI. We used a custom toolbox for processing of MR data (QMRITools for Mathematica -mfroeling.github.io/QMRITools). Two of the authors (MF and LAMO, 13 years and 3 years of experience, respectively) checked data for motion artifacts and quality. We reconstructed DIXON data with an IDEAL method with estimation of B0 and T2*, T2-mapping data with extended graph (EPG) fitting [22] and DTI data with iWLLS method with REKINDLE outlier detection. We used principle component analysis (PCA) for denoising and for obtaining the signal to noise ratio (SNR) of DTI data and corrected for subject motion and eddy current distortion [23].

For a detailed description of the processing steps we refer to previous work [18, 21].

### Comparison of imaging stack

The field of view (FOV) comprised 12 individual muscles per leg; adductor magnus, adductor longus, rectus femoris, the three vasti muscles, semimembranosus, semitendinosus, biceps femoris (long and short head) and the sartorius and gracilis muscle. One of the authors (LAMO) performed manual segmentation of these muscles for each leg with ITK-SNAP [24]. (version 3.6) and at each time-point. As positioning of patients may slightly vary between scans, we used multiple converting steps for alignment to ensure evaluation of the same muscle location at each time-point [14]. In short, we used the registration of the DIXON water image of each of the scans for combined rigid, affine and b-spline registration to the other two time-points (in the assumption that each participant had a total of three scans). The manual segmentation, or mask, at each time-point was transformed accordingly. We took the union of the transformed segmentation and the native segmentation for each time-point. We then repeated this step, where each time-point served as reference and starting point for the converting steps. Finally, only a match between the three segmentations in the image space of those three scans was taken for analysis which assures that we evaluated exactly the same muscle location at all timepoints.

### Statistical analysis

We compared clinical scores and qMRI outcomes with linear mixed effects models. We plotted all outcomes as function of the continuous variable time (in years) to account for variation in timing of scans and estimated the average time trend for qMRI data by incorporating a random intercept and slope for time per subject, as well as a random intercept for muscle. Also, we clustered the muscles in a muscle group (hamstrings, adductors, quadriceps). The values at individual muscle level are the mean of all incorporated slices after comparison of imaging stacks between time-points.

The fixed part of the model contained the baseline score and an effect for time. We used the Wald statistic to determine significance (threshold set at *p* < 0.05).

We will refer to patients who were still able to walk as ‘walkers’ and patients who had lost or never acquired the ability to walk as ‘sitters’ (i.e. both type 2 and non-ambulant type 3). Due to the limited sample size, we use only descriptive statistics for the findings in clinical subgroups. We used SPSS for statistical analysis (version 25 for Windows, SPSS Inc. Chicago) and ggplot2 function in RStudio (version 1.3.959, RStudio PBS) for the figures. All source codes and data are available upon request.

## RESULTS

### Study population

**Fig. 1:**
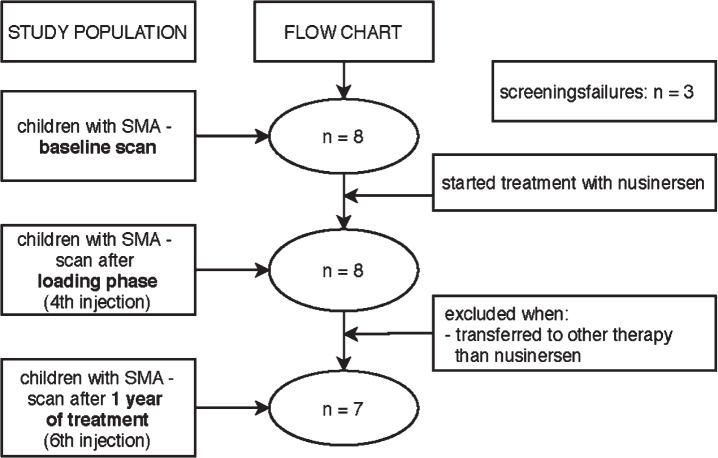
Flowchart of study procedure.

We had to exclude one participant after the 4th injection since he/she wanted to participate in a clinical trial (see flowchart in Fig. 1). All other 7 participants continued nusinersen treatment.

### Clinical evaluation

Clinical scores are presented in Table 2. HFMSE scores were higher at 5th injection compared to baseline in four out seven children. The mean change of the HFMSE score after 6 months of treatment was +1.6 points (95% CI –0.20 to 3.50, *p* = 0.084) [25].

**Table 2:** Clinical parameters over time

Clinical measurements	*n*	Baseline (SE)	Slope / year [95% -CI]	*p*-value
HFMSE	23	31.8 (0.7)	+1.6 [–0.2–3.5]	0.084
MRC sum score	20†	130.8 (1.4)	+5.4 [0.7–10.1]	0.028*
MRC sum score upper leg	23	18.5 (0.4)	+0.3 [–0.7–1.3]	0.504
HHD (N)
Adductors	40	21.8 (0.9)	+6.8 [3.3–10.3]	<0.001*
Hamstrings	46	22.2 (1.9)	+8.9 [4.5–13.3]	<0.001*
Quadriceps	40	4.3 (1.6)	+1.0 [–2.4–4.3]	0.557

Legend table 2: * = *p* < 0.05; † = missing data; CI = confidence interval; HHD = hand-held dynamometry; HFMSE = Hammersmith Functional Motor Scale Expanded, score range 0–66; MRC = Medical Research Council, sum score range 44–210, sum score upper leg range 6–30; N = Newton; *n* = number of observations included in analysis; s = second; SE = standard error.

We could not obtain full MRC scores of 2 patients at two visits because of fatigue. We therefore only obtained MRC scores of muscle groups of the thigh. At the group level, the MRC sum score increase was +5.4 points during the first year of treatment (95% CI 0.70 to 10.10, *p* = 0.028). At individual level, the range of MRC sum change was 0 to 11 points (i.e. 1 subject remained stable). The change in MRC sum scores of thigh muscles after one year was not significantly different from baseline (+0.3, 95% CI –0.70 to 1.30, *p* = 0.50) but we did detect variability between subjects; MRC sum score changes of the thigh varied from a decrease of 1 point (2 subjects), stable (1 subject), to an increase of 1 (3 subjects) or 2 points (1 subject).

Quantitative muscle strength (as measured by HHD) of adductors and hamstrings increased significantly in the course of one year (+6.8 N 95% CI 3.3 to 10.3, baseline 21.8, *p* < 0.001 and +8.9 N 95% CI 4.5 to 13.3, baseline 22.1, *p* < 0.001, respectively). HHD of quadriceps was 4.3 N at baseline and showed no significant change (+1.0 N 95% CI –2.4 to 4.3, *p* = 0.56)

### MR acquisition and processing

To minimize study burden for the subjects, we scheduled MR scans around their hospital visits. We obtained baseline scans the day before (7 patients) or one month before (1 patient) the first administration of nusinersen. We performed the second scan one day prior to the 4th injection (1 patient); on the day of the 4th injection (4 patients) or in the period following the 4th injection (2 patients; 2 weeks since and 5 months, respectively) and the third scan on the day of the 6th injection in 3 patients, after 1 month (2 patients) or 2 months (2 patients) following the 6th injection. We excluded one patient after the 4th injection because of his/her wish to participate in a clinical trial with another drug.

None of the datasets had to be excluded for motion artifacts or data quality, resulting in a total of 25 datasets. 90% of muscles could be segmented, whilst extensive fatty degeneration precluded segmentation in the remainder (Table 3).

**Table 3:** Quantitative parameters over time

qMRI parameter	*n*	Baseline (SE)	Slope / year [95% -CI]	*p*-value
Fat fraction (%)	499	44.2 (0.3)	+3.2 [0.9–5.5]	0.015*
Adductors		43.7 (1.3)	+1.5 [0.3–2.7]	0.014*
Hamstrings		44.0 (0.2)	+3.3 [1.3–5.4]	0.652
Quadriceps		44.5 (0.5)	+5.8 [2.7–8.9]	0.003*
Volume (cm^3^)	502	12.3 (0.2)	+1.2 [–0.4–2.9]	0.133
Adductors		12.6 (0.1)	+0.7 [–0.7–2.2]	0.263
Hamstrings		12.4 (0.2)	+1.2 [–1.1–3.6]	0.293
Quadriceps		11.9 (0.4)	+1.5 [–1.0–4.0]	0.194
Contractile volume (cm^3^)	502	6.8 (0.2)	+0.5 [–0.6–1.6]	0.343
Adductors		7.0 (0.2)	+0.4 [–0.8–1.6]	0.498
Hamstrings		6.9 (0.2)	+0.7 [–1.2–2.6]	0.399
Quadriceps		6.7 (0.1)	+0.0 [–0.9–0.9]	0.927
WaterT2 (ms)	497	26.76 (0.24)	–0.72 [–2.51–1.06]	0.293
Adductors		27.00 (0.59)	–1.28 [–4.06–1.51]	0.299
Hamstrings		29.93 (0.40)	–0.19 [–1.48–1.11]	0.745
Quadriceps		26.61 (0.22)	–0.84 [–2.17–0.49]	0.176
MD (10^–3^ mm^2^/s)	493	1.58 (0.02)	–0.05 [–0.12–0.02]	0.130
Adductors		1.65 (0.05)	–0.03 [–0.20–0.15]	0.714
Hamstrings		1.59 (0.04)	–0.09 [–0.16 ––0.03]	0.007*
Quadriceps		1.57 (0.07)	–0.03 [–0.13–0.07]	0.597
FA	493	0.36 (0.01)	–0.02 [–0.04–0.01]	0.110
Adductors		0.35 (0.01)	–0.03 [–0.07–0.01]	0.133
Hamstrings		0.35 (0.01)	–0.02 [–0.04–0.00]	0.084
Quadriceps		0.36 (0.01)	–0.01 [–0.05–0.02]	0.410
AD (10^–3^ mm^2^/s)	493	2.19 (0.04)	–0.11 [–0.22–0.01]	0.061
Adductors		2.30 (0.06)	–0.10 [–0.39–0.19]	0.431
Hamstrings		2.16 (0.07)	–0.19 [–0.29 ––0.09]	<0.001*
Quadriceps		2.19 (0.08)	–0.06 [–0.19–0.08]	0.405
RD (10^–3^ mm^2^/s)	492	1.04 (0.02)	–0.03 [–0.10–0.04]	0.386
Adductors		1.06 (0.03)	+0.04 [–0.09–0.17]	0.468
Hamstrings		1.06 (0.04)	–0.06 [–0.14–0.01]	0.089
Quadriceps		1.01 (0.03)	–0.02 [–0.11–0.06	0.487

Legend table 3: * = *p* < 0.05; AD = axial diffusivity; CI = confidence interval; FA = fractional anisotropy; mm = millimeter; MD = mean diffusivity; ms = millisecond; *n* = number of observations included in analysis; RD = radial diffusivity; s = second; SE = standard error.

### Muscle fat fraction changes in the course of one year (DIXON)

**Fig. 2:**
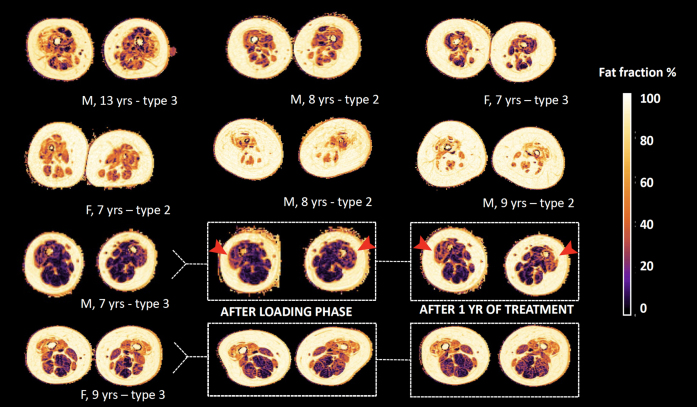
Status of fat infiltration at baseline of participants. Legend: M = male, F = female, yr(s) = year(s). Overview of the status of fat infiltration of all participants, as measured by the DIXON sequence; in the bottom two rows we highlight the three consecutive scans of two subjects. Note the visible change of color in the anterior compartment of one male subject, as indicated by the red arrows, that indicates progression of fat infiltration.

**Fig. 3:**
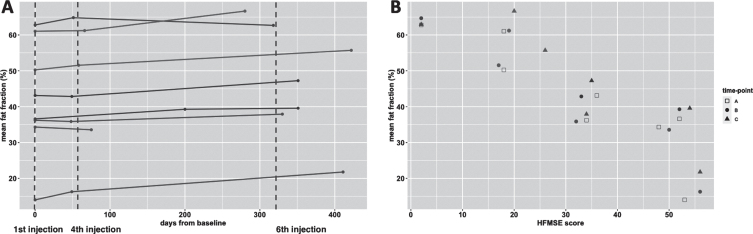
Fat fraction over time and relation with motor function score. Legend: HFMSE = Hammersmith Functional Motor Scale, Expanded. Panel A presents the trajectory of fat fraction of subjects over time, the timing of each MR scan is presented as a bullet. The vertical dotted line indicates the schedule of the 1st, 4th and 6th nusinersen injection. In panel B the timing of scans is presented as A (baseline); B (after loading phase) and C (one year on treatment) and fat fraction at each time-point is presented in relation to the score on the HFMSE scale. Subjects go by the same color in the panels.

Figure 2 depicts the cross-sectional image of fat infiltration of thigh muscles of each participant at baseline. Table 3 summarizes quantitative MR data over time. The average fat fraction of participants was 44.2% (95% CI 43.5 to 44.9 at baseline). Quantification of fat infiltration showed an increase of 3.2% in fat fraction during treatment (95% CI 0.9 to 5.5, *p* = 0.015). Figure 3 (panel A) shows the slope of fat fraction of individual patients over time, and their relation with motor function scores (panel B).

Fat infiltration exceeded 40% in all muscles at baseline, except for the semimembranosus and the adductor longus. Notably, the adductor longus had the lowest fat fraction, which decreased in the course of one year (21.5% to 19.4%). The highest increase in fat fraction was in the rectus femoris (+10%) and lowest in the sartorius (+1.5%) after one year. At the level of muscle groups, the quadriceps (consisting of the rectus femoris and vasti muscles) showed the greatest fat increase; +5.8% (95% CI 2.7 to 8.9, baseline 44.5, *p* = 0.003). The fat fraction of the adductor muscle group (adductor magnus and longus muscle) increased by 1.5% (95% CI 0.3 to 2.7, *p* = 0.014), and that of hamstrings (semimembranosus, semitendinosus and biceps femoris) by +3.3% (95% CI 1.1 to 5.5, *p* = 0.65). We did not find a significant decrease in contractile volume over time (Table 3). At baseline and after the 6th injection, fat fraction negatively correlated with contractile volume of thigh muscle (R = –0.25, *p* = 0.001 and R = –0.32, *p* < 0.001, respectively). However, the change in fat fraction did not correlate with a change in contractile volume (R = –0.15, *p* = 0.086).

### Fat fraction in relation to clinical characteristics

**Fig. 4:**
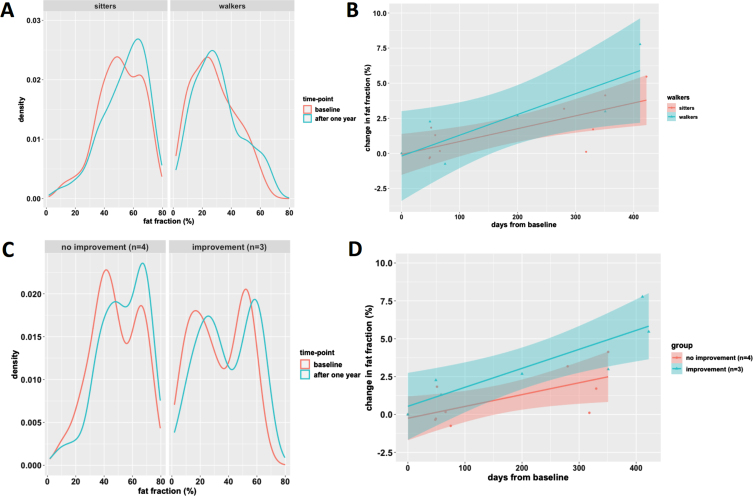
Plots of fat fraction over time for walkers vs. sitters and motor function improvement group. The top row (A-B) presents the fat fraction when subjects are classified upon ambulatory status at baseline as ‘sitters’ or ‘walkers’. Note the lower average fat fraction in walkers (panel A). Furthermore, the 95% confidence interval (shaded area) of change in fat fraction of the groups mostly overlaps. The bottom row (C-D) presents the fat fraction when subjects are further subclassified based on their motor function score change. Note the bimodal fat distribution in responders panel A, and the seemingly steeper slope of change in fat fraction of responders.

Three patients were walkers and 5 were sitters (1 subject with type 3 and 4 with type 2). Figure 4 shows the histogram and slope of fat infiltration for both groups. Walkers had an lower average fat fraction of thigh muscles at baseline than sitters (baseline 28.2% versus 50.7%; +2.6% versus +3.0% over time, respectively). In parallel, we observed a decrease (between 1.3 to 5.8%) in fat fraction in walkers in the adductor longus, the adductor magnus, semimembranosus and semitendinosus. Walkers had a higher HFMSE score at baseline.

QMRI results of three children with motor function improvement showed the most pronounced change in fat fraction. The fat fraction distribution of this group was bimodal with low and high fat infiltration peaks (Fig. 4C). This pattern of two peaks persisted and evenly shifted towards higher fat infiltration in the course of one year. In contrast, the fat distribution of thigh muscles of the other children did not show a bimodal distribution, but a broad slope from moderate to high (50 to 70%) fat fraction. At baseline, the fat fraction of children with the most pronounced motor function improvement was lower compared to children with modest or no motor function change (33.6% vs 50.8%). However, increase in fat fraction in the course of one year was greater (+5.4% vs. +2.4%) (Fig. 4D).

### Quantitative MR parameter:waterT2

T2 imaging had a slope of –0.7 ms (95% CI –0.2 to 0.1, baseline 26.7 ms) in the course of one year. The changes compared to baseline were not significant (*p* = 0.29) either on the group level or for individual muscle groups.

### Quantitative MR parameters: MD, FA, AD and RD

We observed a non-signifcant negative slope for all DTI parameters MD, FA, AD and RD when we performed combined analysis of all leg muscles (Table 3). The negative slopes of MD and AD seem mainly driven by a significant decrease seen in the hamstrings (slope MD: –0.1 mm^2^/s/year, *p* < 0.01 and slope AD: –0.19 mm^2^/s/year, *p* < 0.001, respectively), while values did not change significantly in quadriceps and adductors. FA showed the strongest negative decrease for the hamstrings (–0.02/year, *p* = 0.084), but similar to RD not for all muscle groups.

## DISCUSSION

In this study we describe quantitative MR parameter changes in thigh muscles of 8 children with SMA aged 7 to 13 years during one-year treatment with nusinersen. Segmentation of individual muscles allowed comparison of longitudinal MR data for the monitoring of treatment effects.

The DIXON and DTI suggest simultaneous and divergent processes. We observed continued fatty degeneration of muscle groups that were already affected at the start of treatment. In parallel, DTI results may reflect improvements in the microstructure of muscle tissue. Normalization of some DTI values co-occuring with fatty degeneration may seem contradictory. However, distinct pathological processes can vary between muscle groups, as seen in DMD with fat infiltration and accompanying muscle hypertrophy [26]. Together, these alterations differ from the changes observed in treatment-naïve adult patients in whom the natural disease progression is characterized by progressive fatty degeneration at the loss of contractile muscle tissue [14]. Our data show that although progression of fatty infiltration of thigh muscles is also present in young children with SMA who are treated, contractile volume of thigh muscles remains stable.

Fatty replacement increased despite treatment, in line with previous observations [27], although the rates at which this happened differed between muscle groups, possibly reflecting differences in vulnerability of muscle groups. The quadriceps, which is a relatively weak muscle group in SMA, [18, 28–30] showed the highest one-year increase in fat, followed by the hamstrings, whilst the anatomy and function of the adductors was relatively preserved. The adductor longus was the only muscle in which average fat content decreased during treatment in all children. This is consistent with observations in adults by Savini et al. and by Gallone et al. [27, 31].

The gains in HFMSE scores that we observed in a subgroup of children indicate that treatment success is not explained by a reverse of fatty degeneration [31, 32]. This observation is consistent with other publications of fat fraction changes in upper leg muscle during nusinersen treatment. The work by Gallone and by Sprenger et al. showed a non-significant increase of approximately 2% in fat fraction after 14 months, and stable fat measures over 6 months, respectively. Despite the difference in methodology and adult patient population, their and our work converge in the sense that clinical improvement occurs without a reverse of muscle fat infiltration.

The association of DIXON method with clinical scores has been described [31], as per a study by our group in which we even established disease progression with DIXON ahead of clinical changes [33]. In this treatment cohort, seemingly counter-intuitive, the greatest increase in fat fraction appears to occur in patients with lower fat fraction at baseline and with the most pronounced motor function improvement. In addition, we observed a specific pattern of fat distribution in children who experienced gains in motor function scores. This bimodal distribution of fatty infiltration may therefore hold biomarker value. We have previously observed a similar bimodal pattern in some adults [14, 31, 33, 34]. This bimodal pattern most likely indicates the presence of muscles that may still respond to treatment (i.e. low levels of fat;<30%), which in turn adjusts towards lower fat fraction means. Our previous work on disease progression suggests that fat degeneration occurs at a faster pace in low ranges of fat fractions and slows down towards end-stage fat infiltration [14]. Therefore, the value of bimodal fatty distribution as qMRI biomarker deserves further study.

Diffusion tensor imaging provides the directional properties of water diffusion. The relation between each of the directional parameters can be used to probe tissue microarchitecture [35]. For example, the finding of lowered average diffusion in a increased anisotropic medium was an indication of shrinkage of the cellular compartment, i.e. muscle atrophy, [36] as we established in a previous study on thigh muscles in patients with SMA [18].

The mean diffusivity (MD) at baseline was higher than measured in adult patients with SMA [18], probably due to lower levels of fat infiltration in children, [37, 38] but still lower compared to values known of healthy muscles (i.e. 1.4 to 1.8) [21, 39]. MD values are in line with what can be expected based on the range of MD lower fat fractions regions, based on a model of cross-sectional data in our previous study [33].

Previously, we demonstrated the relation between FA and thigh muscle strength [18]. Fractional anisotropy (FA) at baseline was high compared to findings in healthy muscles. After start of nusinersen treatment, we observed significant changes towards normal DTI values in hamstrings with similar trends in other muscle groups. A decrease in FA is explained by an axial diffusivity (AD) decrease, while radial diffusivity (RD) remains constant. This means that during treatment diffusion restriction in axial directions increases, possibly implicating that atrophy is gradually reversed [40]. The trend of normalization of MD, RD and AD values could reflect a decrease of membrane permeability, or an increase of intracellular actin and myosine [41]. We hypothesize that these changes indicate normalization of muscle microstructure. To date, one other study explored DTI indices during nusinersen treatment, but comparison is hampered by the lack of formal statistical testing as it concerns a case-study of two patients [42]. Taken together, our data suggest that treatment effects could manifest in the restoration of structurally abnormal but viable muscle tissue, rather than in a decrease of fatty infiltration of muscle tissue.

We found no evidence for relevant wT2 changes that accompany fat infiltration. T2 mapping has often been used as meaningful biomarker in other neuromuscular diseases but seems not useful in SMA on account of three subsequent studies [14, 18, 22].

The sample size is a limitation of this study. However, the analysis of individually segmented muscles increased statistical power. However, results are predominantly presented per muscle group, given the extent of quantitative MR parameters, this did not compromise results. Despite extensive fatty replacement, we were able to include muscles with up to 80% fat fraction in our final analysis. Not surprisingly, muscle MRI is most informative when applied in relatively preserved muscle tissue, both in children and adults [14]. Although for practical reasons we opted for the inclusion of children from the age of 6 years, age itself is not a limitation for the use of MR imaging in a clinical or scientific setting, although the additional need for sedation of younger children requires careful consideration.

Since we did not include a matched group of control subjects, patients served as their own reference. Theoretically, the observed changes in DTI parameters could reflect natural history rather than treatment effects. This is unlikely, since the increase of fat replacement is similar to observations in adults and natural history of children from the age of 6 years on is characterized by slowly progressive decline in muscle strength [13]. Given this expected functional decline, we believe that contractile muscle remaining stable in these children is rather due to treatment than to growth or maturation. We therefore think it is highly unlikely that pathophysiological processes captured by qMRI differ between children and adults. Larger sample sizes and a reference cohort will be almost impossible to obtain now that treatment has become part of standard care.

Lastly, we deliberately refrained from using the term ‘treatment responders’ and opted for an objective description of clinical outcomes as study design and sample size did not lend themselves for treatment efficacy evaluation.

To conclude, quantitative MR parameters of the DIXON and DTI sequence provided insight into parallel ongoing fatty infiltration with possible microstructural normalization of thigh muscles in young patients during their first year of nusinersen treatment. Quantitative MRI could serve as a biomarker for treatment effects at the tissue level and possibly even to identify responders.

## ABBREVIATIONS

AD: axial diffusivity
DMD: Duchenne Muscular Dystrophy
DTI: diffusion tensor imaging
FA: fractional anisotropy
FOV: field of view
HFMSE: Hammersmith Functional Motor Scale, Expanded
HHD: hand-held dynamometry
MD: mean diffusivity
MRC: Medical Research Council
PCA: principal component analysis
qMRI: quantitative MRI
RD: radial diffusivity
SMA: spinal muscular atrophy
SMN: survival motor neuron
SNR: signal to noise ratio
SE-EPI: spin-echo echo planar imaging
SPAIR: spectral attenuated inversion recovery
SPIR: spectral presaturation with inversion recovery
